# Correlation between uterine fibroids with various magnetic resonance imaging features and therapeutic effects of high-intensity focused ultrasound ablation

**DOI:** 10.12669/pjms.314.7294

**Published:** 2015

**Authors:** Hailing Cheng, Chen Wang, Jun Tian

**Affiliations:** 1 Hailing Cheng, Huaihe Hospital of Henan University, Kaifeng 475000, P. R. China; 2 Chen Wang, Huaihe Hospital of Henan University, Kaifeng 475000, P. R. China; 3 Jun Tian, Huaihe Hospital of Henan University, Kaifeng 475000, P. R. China

**Keywords:** High-intensity focused ultrasound ablation, Uterine fibroid, Magnetic resonance imaging, Therapeutic effect, Correlation

## Abstract

**Objective:**

To explore the correlation between magnetic resonance imaging (MRI) features of uterine fibroids (UFs) and therapeutic effects of high-intensity focused ultrasound ablation (HIFUA), and to provide evidence for UFs diagnosis with MRI in clinical practice.

**Methods:**

Forty-three UFs patients who were treated in our hospital from April 2012 to June 2014 were selected, including 72 UFs (48 multiple and 24 single UFs). Transverse, sagittal and coronal MRI scanning was performed one week before and after HIFUA to record UF number, location, type (intramural fibroid, submucosal fibroid and subserosal fibroid), mean diameter, hemoperfusion state, volume and ablation rate. The patients were followed up in the postoperative 1st, 2nd and 3rd months.

**Results:**

HIFUA exerted the best ablative effect on fibroids on the anterior uterine wall (F=26.763, P=0.036). Various types of fibroids were ablated significantly differently (F=3.406, P<0.05) by HIFUA that was most effective for ablating the subserosal ones. Having significantly different ablative effects on UFs with different radial line lengths (F=29.94, P<0.05), HIFUA ablated those with radial line lengths of 3-5 cm most effectively. For UFs with different T2WI signal intensities, HIFUA also functioned significantly differently (F=3.179, P=0. 03).

**Conclusion:**

HIFUA exerted significantly different ablative effects on UFs with various MRI features. Therefore, these features were well correlated with the therapeutic effects of HIFUA, allowing MRI as a promising diagnostic protocol.

## INTRODUCTION

As the most common benign gynecological tumor (over 50% of all cases), uterine fibroid (UF) is now threatening 30% of the women of childbearing age.^1^ UF burdens patients both psychologically and physiologically by shortening menstrual cycle, prolonging menstrual bleeding and even leading to infertility.^2^ Traditionally, UFs are treated by hysterectomy which minimizes recurrence risk in the price of fertility deprivation,^3^ so this method is not acceptable among young females. Although minimally invasive or non-invasive surgeries (e.g. laparoscopic myomectomy, vaginal hysterectomy with cystectomy and uterine artery embolization) have been used to retain the uterus, they have not been widely applied due to high recurrence rate and treatment cost.^3^ High-intensity focused ultrasound ablation (HIFUA), on the other hand, has been applied in clinical practice for over 10 years as a non-invasive method that retains the uterus without inducing obvious side effects. When the pathological data of patients are unavailable, the therapeutic effects of HIFUA are mainly evaluated by using ultrasonic imaging, enhanced computed tomography, magnetic resonance imaging (MRI) and positron emission tomography.^4^

In this study, patients with UFs were examined by MRI before and after HIFUA, aiming to analyze the therapeutic effects of this method on UFs and to provide theoretical evidence for MRI in preoperative evaluation, intraoperative guidance and postoperative assessment.

## METHODS

### Subjects

Forty-three UFs patients who were treated in our hospital from April 2012 to June 2014 were selected, including 72 UFs (48 multiple and 24 single UFs). They were aged 25-48 years old, with the average of (38.34±5.31). This study had been approved by the ethic committee of our hospital, and written consent has been obtained from all groups.

### Inclusion criteria

(1) In accordance with the diagnostic criteria of UFs; (2) non-menopausal patients who desired to retain the uterus not for childbirth; (3) non-pedunculated subserosal fibroids with the maximum diameter of over 2 cm; (4) psychologically normal patients who were able to communicate and to describe their feelings.

### Exclusion criteria

(1) Patients with cervical or vascular smooth muscle fibroids; (2) patients with vaginitis, pelvic inflammation and other gynecological diseases; (3) patients with suspected pelvic tissue and organ adhesions; (4) patients with heart disease or pacemaker insertion; (5) pregnant and lactating women.

### MRI procedure

Transverse, sagittal and coronal MRI scanning was performed one week before and after HIFUA with the same scanning sequence and parameters by using Achieva3.0T dual gradient superconducting magnetic resonance imaging device and GESignaHDxt1.5T superconducting magnetic resonance imaging device (Philips, Netherlands).

### HIFUA procedure

HIFUA was performed with JC200 Focused Ultrasound Tumor Therapeutic System (Chongqing Haifu Medical Technology Co., Ltd.). The main parameters included: power of 100-400 W, biological focal region of 3 mm × 3 mm × 8 mm, probe frequency of 0.8 MHz, focal length of 132 cm, gas content of medium water of <3 × 10^-6^, and ultrasound contrast agent of sulfur hexafluoride microbubbles.

### Indices for evaluation

UF location, type (intramural fibroid, submucosal fibroid and subserosal fibroid), mean diameter, volume, T2WI signal intensity and ablation rate were recorded.

### Statistical analysis

All data were analyzed by SPSS17.0 and expressed as x±s. UF location, T2WI signal intensity, average radial line length and ablative response were subjected to univariate F test. The differences with P<0.05 were considered statically significant and compared by SNK-q test.

## RESULTS

### HIFUA outcomes for different types of UFs

**Fig.1:**
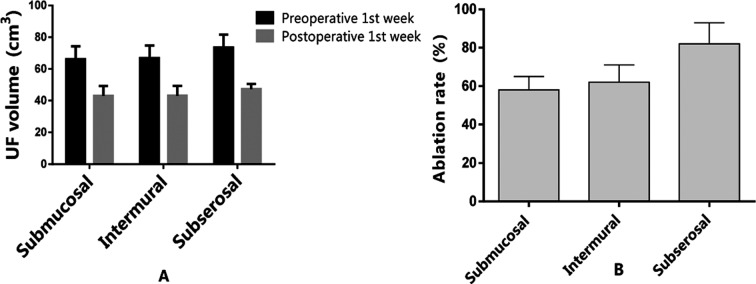
Ablative effects of HIFUA on different types of UFs.A: UF volumes one week before and after HIFUA; B: ablation rates of different types of UFs.

**Fig.2:**
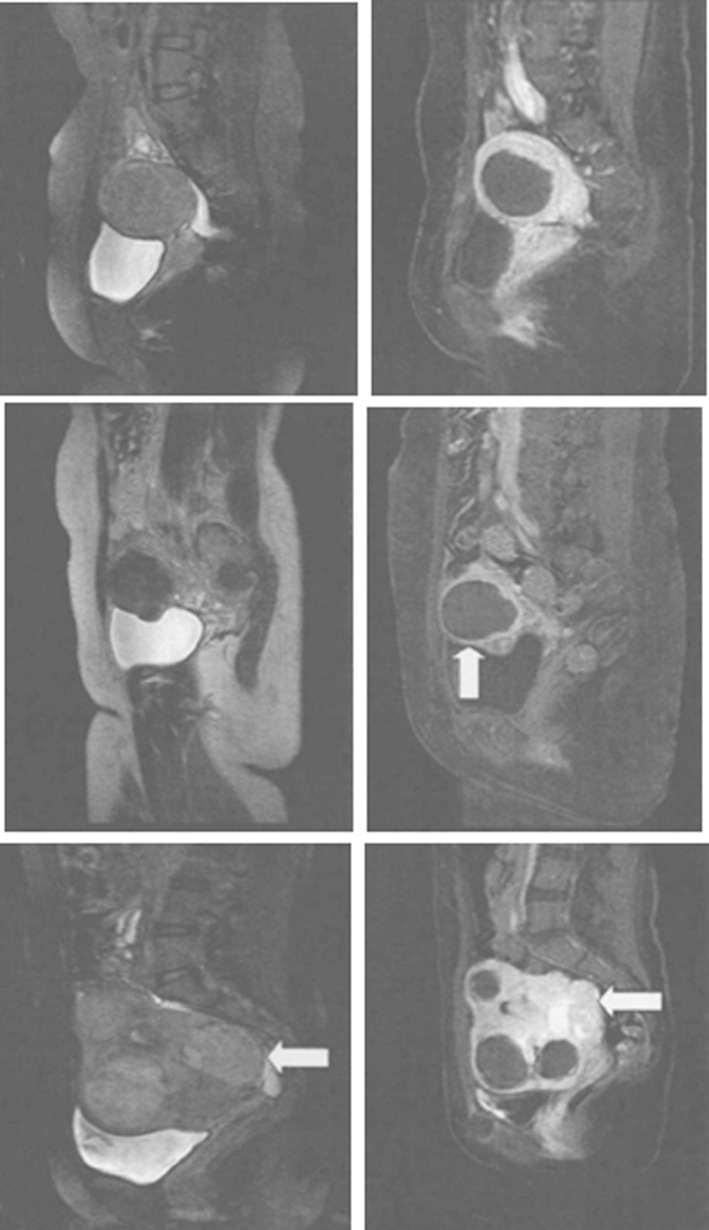
MRI images for different types of UFs. Left: Before HIFUA; right: after HIFUA. First row: intramural fibroid; second row: subserosal fibroid; third row:submucosal fibroid.

The volumes of different types of tumors were significantly decreased after HIFUA compared with those before (P<0.05) (Fig.1), i.e. all of them were well treated. Various types of fibroids were ablated significantly differently (F=3.406, P<0.05) by HIFUA. As evidenced by SNK-q test, submucosal and intramural fibroids had similar ablation rates (P>0.05), which were both significantly lower than that of the subserosal ones (P<0.05). The MRI images before and after HIFUA are shown in Fig.2.

### HIFUA outcomes for UFs with different locations

**Fig.3:**
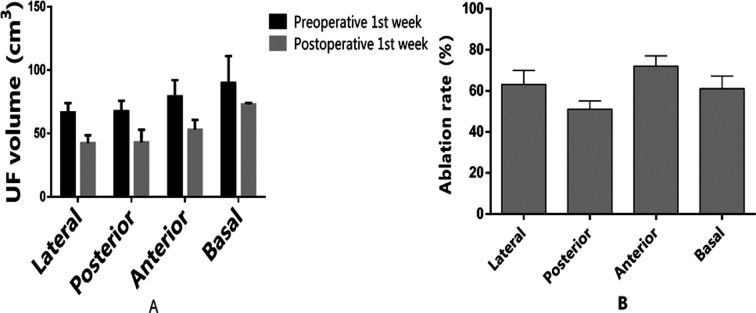
Ablative effects of HIFUA on UFs with different locations. A: UF volumes one week before and after HIFUA; B: ablation rates of UFs with different locations.

**Fig.4:**
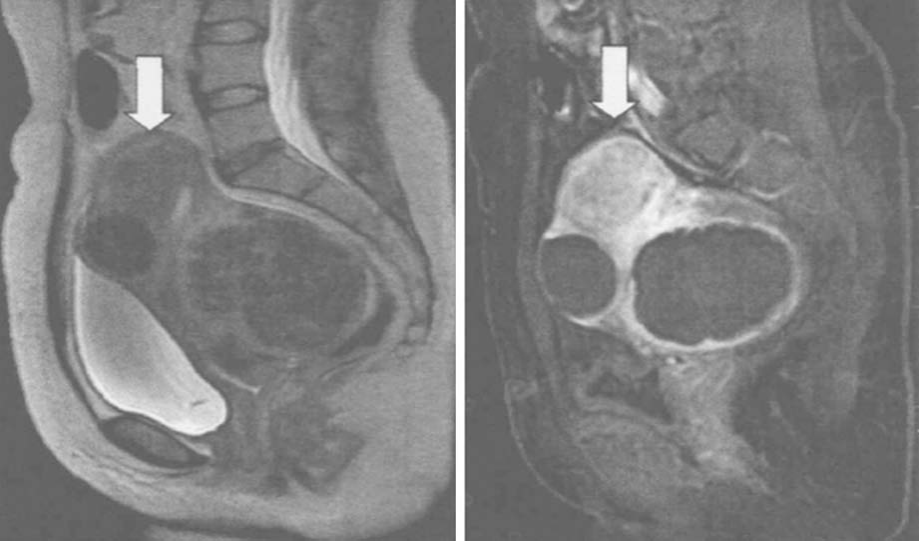
MRI images for UFs with different locations. Left: Before HIFUA; right: after HIFUA.

As shown in Fig.3, UFs with different locations are all significantly shrunken by HIFUA (P<0.05). Univariate F test showed these UFs had significantly different ablation rates (F=26.763, P=0.036). Meanwhile, SNK-q test exhibited that HIFUA exerted the best ablative effect on fibroids on the anterior uterine wall, which significantly surpassed those on the lateral, posterior and basal walls (P<0.05). The MRI images before and after HIFUA are shown in Fig.4.

### HIFUA outcomes for UFs with different radial line lengths

The volumes of UFs with different radial line lengths were all significantly reduced by HIFUA (P<0.05) (Table-I). Univariate F test showed that HIFUA ablated those with radial line lengths of 3-5 cm most effectively (F=29.94, P<0. 05), and SNK-q test showed that the ablation rates of UFs with the other three radial line lengths were similar (P>0.05), which were significantly inferior to that of UFs with the lengths of 3-5 cm (P<0.05) (Table-II).

**Table-I:** Ablation of UFs with different radial line lengths.

Radial line length	Preoperative volume (cm^3^)*	Postoperative volume (cm^3^)**	Ablation rate (%)***
<3	2.49±2.4	2.1±1.7	48±25
3-5	32.16±14.8	20.8±14.7	76±3
5-7	112.27±23.4	68.27±27.9	56±10
>7	167.25±103.1	25.05±9.5	63±39

* F=246. 85, P<0.05,

** F=45. 138, P<0.05,

*** F=29. 94, P<0.05.

**Table-II:** SNK-q test results for the ablation rate of UFs with different radial line lengths.

Radial line length	1	2
<3	0.4769	
>7	0.6343	
5-7	0.6453	
3-5		20.9746
Sig	0.987	1.000

### Time-dependence of UF volume and reduction rate

**Fig.5:**
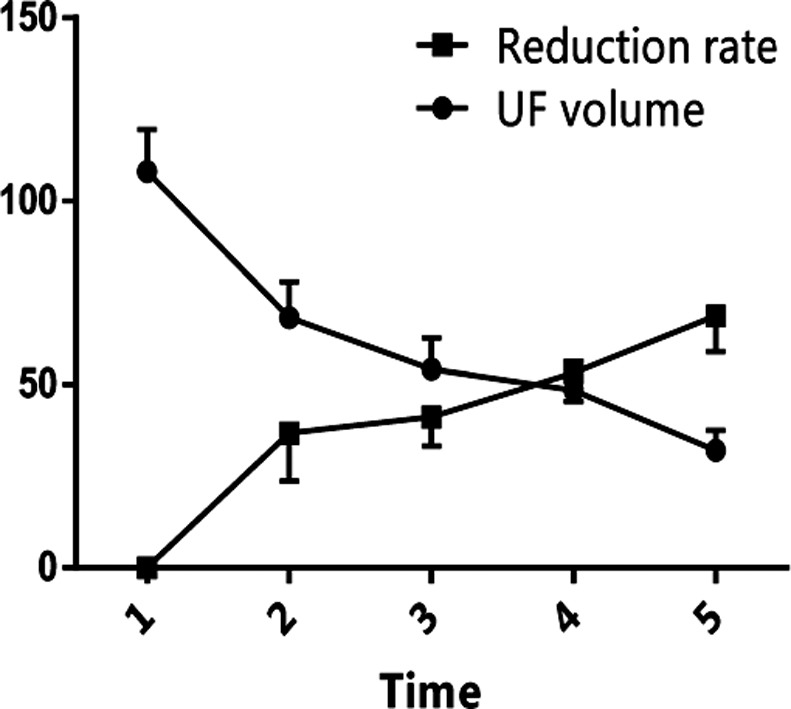
Time-dependence of UF volume and reduction rate. 1: Preoperative 1st week; 2: postoperative 1st week; 3: postoperative 1st month; 4: postoperative 2nd month; 5: postoperative 3rd month.

With elapsed time, the average UF volume gradually decreased from (108±11.6) cm^3^ one week before surgery to (32.03±5.49) cm^3^ in the postoperative 3rd month (Fig.5), whereas the reduction rate increased from (36.75±13.12)% in the postoperative 1st week to (68.74±9.78)% in the postoperative 3rd month.

### HIFUA outcomes for UFs with different T2WI signal intensities

The ablation rates of UFs with low T2WI signal, equisignal, high signal and mixed signal intensities were (71.05±6.2)%, (46.86±16.04)%, (56.12±14.32)% and (62.12±28.13)% respectively. For UFs with different T2WI signal intensities, HIFUA functioned significantly differently (P<0.05) (Table-III).

**Table-III:** Ablative effects of HIFUA on UFs with different T2WI signal intensities.

T2WI signal	n (%)	Preoperative volume (cm^3^)*	Postoperative volume (cm^3^)**	Ablation rate (%)***
Low signal		68.29±8.35	42.12±9.0	71.05±6.2
Equisignal		69.91±17.2	45.12±9.8	46.87±16.04
High signal		99.45±9.6	57.12±5.3	56.12±14.32
Mixed signal		64.45±7.56	42.15±7.34	62.12±28.13

* F=2. 015, P=0. 121,

** F=1.220, P=0. 310,

*** F=3. 179, P=0. 03.

## DISCUSSION

The treatment outcomes of UFs remain unsatisfactory owing to high incidence and limitation of traditional protocols. The merit of HIFUA in UF treatment has been well documented,^5^ but there remains controversy over the method for dynamic intraoperative monitoring. In this study, MRI scan was performed one week before and after surgery and in the postoperative 1st, 2nd and 3rd months to analyze the correlation between MRI features of UFs and therapeutic effects of HIFUA.

HIFUA exerted the best ablative effect on fibroids on the anterior uterine wall, probably because ultrasound attenuated through different channels encountering different acoustic impedances during propagation in tissues.^6^ Fibroids on the anterior uterine wall, which are mainly surrounded by bladder muscle and normal uterine tissues, are distant from the intestinal tract and the sacrum. Therefore, HIFUA can be conducted safely and comfortably, accompanied by high ultrasonic intensity due to shallow location.^7^,^8^ In contrast, UFs on the posterior wall, which are close to the sacrum and surrounded by a large number of nervous tissues, can only be ablated painfully with severe ultrasonic attenuation. Meanwhile, low treatment efficiency is accompanied by increasing odds of skin burns and blisters.^9^,^10^ Moreover, the ablation dosage must be increased owing to long distance from the skin surface and long ablation time, which may easily lead to complications.

UFs comprise dense bundles of smooth muscle cells and various amounts of fibrous connective tissues, which are generally classified into common, cellular and degeneratedtypes.^11^ The features of MRI signals inside UFs are determined by the proportion of smooth muscle cells to connective tissues, as well as the shape, arrangement, distribution and degeneration of cells.^12^ In general, MRI T1WI signals of UFs are not significantly different, but T2WI signals of foci and the myometrium differ greatly.^13^ To this end, Schwartz et al. have typed UFs according to the features of MRI signals.^14^ In this study, UF type and T2WI signal intensity all significantly affected the therapeutic effects of HIFUA (P<0.05), and UFs were ablated optimally at low signal intensity. Hence, it is possible to predict the ablative effects of this method by simply analyzing preoperative images, providing valuable and reliable evidence.^15^,^16^ Undegenerated UFs usually have low T2WI signal intensities, but large ones are prone to degeneration and experiencing increase of free water content,^17^ which elevates the signal intensity and negatively affects ablation that hinders ultrasonic energy deposition. Furthermore, depending on UF types, the treatment outcomes of HIFUA were indeed correlated with MRI features.

In summary, HIFUA exerted significantly different ablative effects on UFs with various MRI features. Accordingly, these features were well correlated with the therapeutic effects of HIFUA, allowing MRI as a potentially eligible diagnostic protocol.
